# Advances in understanding the mechanisms underlying acquired resistance to third-generation tyrosine kinase inhibitors in non-small cell lung cancer

**DOI:** 10.3389/fcell.2026.1867246

**Published:** 2026-08-24

**Authors:** Gu-Ha A-Lai, Lian Li, Guangzhi Ma, Yong-Sheng Zhao, Peng Yao, Yi-Dan Lin

**Affiliations:** 1 Department of Thoracic Surgery, West China Hospital, Sichuan University, Chengdu, China; 2 Outpatient Department, West China Hospital, Sichuan University, Chengdu, China; 3 Lung Cancer Center/Lung Cancer Institute, West China Hospital, Sichuan University, Chengdu, China

**Keywords:** advances, mechanism, NSCLC, resistance, TKI

## Abstract

Acquired resistance to third-generation epidermal growth factor receptor (EGFR) tyrosine kinase inhibitors (TKIs) presents a formidable challenge in the treatment of non-small cell lung cancer (NSCLC). Despite the remarkable efficacy of these agents, resistance inevitably develops, typically within approximately 10 months of treatment initiation. This review elucidates the multifaceted mechanisms driving this resistance, broadly categorized into on-target EGFR-dependent alterations and off-target EGFR-independent bypass pathway activations. On-target mechanisms include the emergence of tertiary EGFR mutations, most notably C797S, which disrupts TKI binding. Off-target mechanisms encompass the activation of alternative signaling pathways such as MET and HER2/HER3 amplification, as well as histological transformations and complex changes within the tumor microenvironment. Furthermore, recent discoveries highlight the role of epigenetic dysregulation and metabolic reprogramming in fostering resistance. To counter this pervasive adaptability, advanced diagnostic methodologies, including liquid biopsy and high-resolution omics technologies, are crucial for real-time molecular profiling. The field is actively exploring emerging combination therapeutic strategies to circumvent these diverse resistance pathways, aiming to prolong clinical benefits and improve patient outcomes. The persistent emergence of resistance underscores that current targeted therapies, while revolutionary, are primarily disease-modifying rather than curative, necessitating continuous innovation to overcome the inherent biological challenge of tumor adaptability and heterogeneity.

## Introduction: the evolving landscape of EGFR-Mutant NSCLC therapy

Lung cancer remains the leading cause of cancer-related mortality globally, with non-small cell lung cancer (NSCLC) constituting the majority of cases, accounting for 80%–85% (Coudray et al., 2018). A significant proportion of NSCLC, particularly adenocarcinomas, is characterized by oncogenic driver mutations in the epidermal growth factor receptor (EGFR) gene. These mutations are observed in 10%–15% of Western populations and up to 50% of Asian patients, and the most common activating EGFR mutations are small multi-nucleotide in-frame deletions in exon 19 (ex19del) and a point mutation in exon 21, p. Leu858Arg (L858R), collectively representing approximately 90% of detected EGFR mutations (Yang et al., 2018). These alterations lead to constitutive activation of EGFR signaling, promoting uncontrolled cell proliferation and survival, rendering tumors “oncogene addicted” (Li et al., 2025a).

The identification of these activating EGFR mutations revolutionized NSCLC treatment, ushering in the era of targeted therapies. First-generation EGFR TKIs, such as gefitinib and erlotinib, and second-generation TKIs, including afatinib and dacomitinib, demonstrated superior efficacy compared to traditional chemotherapy in patients with EGFR-mutant NSCLC (Masters et al., 2015). However, the clinical benefits of these earlier-generation TKIs were often limited by the inevitable development of acquired resistance, typically occurring within 9–14 months of treatment initiation, while the most prevalent mechanism of this acquired resistance was the emergence of a secondary EGFR mutation, T790M, located in exon 20, which acts as a “gatekeeper” mutation by sterically hindering the binding of first- and second-generation TKIs to the ATP-binding pocket of EGFR (Lu et al., 2012). This mutation accounted for 50%–60% of acquired resistance cases to earlier TKIs (Minari et al., 2016).

To address the challenge posed by T790M-mediated resistance, third-generation EGFR TKIs were developed. Osimertinib stands as the most widely approved agent in this class, designed to selectively and irreversibly inhibit both EGFR-sensitizing mutations (ex19del, L858R) and the T790M resistance mutation by forming a covalent bond with the C797 residue in the ATP-binding pocket (Ercan et al., 2015). Other notable third-generation TKIs include lazertinib, furmonertinib, and almonertinib, which similarly target sensitizing T790M mutations (Nagasaka et al., 2021). Osimertinib has demonstrated significant clinical benefits, including extended progression-free survival (PFS) and overall survival (OS). For instance, the FLAURA trial reported a median PFS of 18.9 months and OS of 38.6 months in untreated patients with EGFR-sensitizing mutations (Soria et al., 2018). Its efficacy extends to the adjuvant setting, as shown by the ADAURA trial, where it achieved an impressive 5-year OS rate of 85% for stage II-IIIA patients (Herbst et al., 2023).

Despite these substantial advancements, acquired resistance to third-generation EGFR TKIs, particularly osimertinib, remains an inevitable clinical reality, typically emerging within approximately 10 months of treatment initiation (Minari et al., 2016). This resistance is characterized by its complex and heterogeneous nature, involving both on-target EGFR-dependent mechanisms and off-target EGFR-independent pathways. A comprehensive understanding of these diverse mechanisms is paramount for the development of effective subsequent therapeutic strategies and for guiding personalized treatment approaches (Schmid et al., 2020). Besides, comprehensive overview of third-generation EGFR TKIs and relative clinical efficacy was summarized in Table 1.

**TABLE 1:** Overview of key third-generation EGFR TKIs and their clinical efficacy.

TKI name	Primary target mutations	Key clinical trial(s)	Median PFS (Relevant Setting)	Key adverse events	Unique features/Advantages
Osimertinib	Ex19del, L858R, T790M	FLAURA, ADAURA, LAURA, AURA	18.9 months (1st-line) (Soria et al., 2018); 9.6–10.1 months (2nd-line, T790M+) (Mok et al., 2017)	Diarrhea, rash, stomatitis, paronychia, ILD/pneumonitis, QTc prolongation, LVEF decrease (Priantti et al., 2024)	Irreversible binding to C797; effective against T790M; approved 1st-line, 2nd-line, and adjuvant (Schmid et al., 2020)
Lazertinib	Ex19del, L858R, T790M	LASER, CHRYSALIS	20.6 months (1st-line monotherapy vs. gefitinib) (Reungwetwattana et al., 2023)	Rash, nail toxicity, infusion reactions, fatigue, stomatitis, nausea, diarrhea, constipation, edema, musculoskeletal pain, ALT/AST elevations (Lee et al., 2022)	High selectivity for sensitizing and T790M mutations; lower risk of skin/cardiac AEs vs. osimertinib; good intracranial activity (Soo et al., 2023)
Furmonertinib	Ex19del, L858R, T790M	FURLONG	20.8 months (1st-line, classical EGFR-mutations) (Ding et al., 2024)	Not detailed in snippets, generally good tolerability reported (Sun and Wang, 2024)	Unrivalled mPFS in unfavorable prognostic factors (L858R, CNS metastasis); effective in complex resistance (Sun and Wang, 2024)
Almonertinib	L858R/T790M	AENEAS	19.3 months (1st-line)	Not detailed in snippets, ILD reported in combination (Yang et al., 2025)	Effective against L858R/T790M; resistance associated with enhanced glycolysis (Wei et al., 2025)

## Mechanisms of acquired resistance to third-generation EGFR TKIs

**FIGURE 1:**
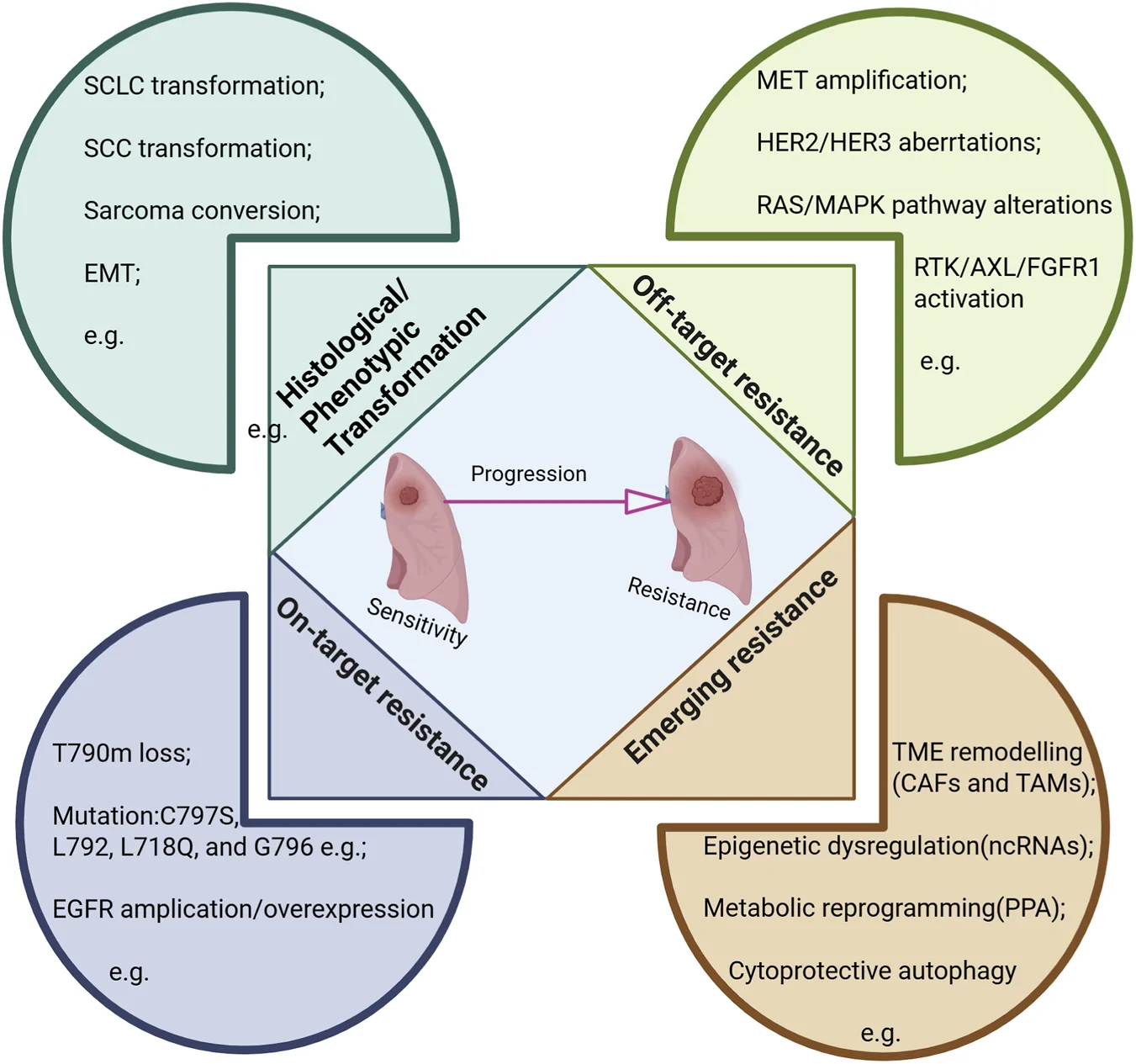
Mechanisms of acquired resistance to third-generation EGFR TKIs.

The development of acquired resistance to third-generation EGFR TKIs is a complex and heterogeneous process, influenced by the line of treatment and the duration of therapy (Gomatou et al., 2023). As demonstrated in Figure 1, Resistance mechanisms are broadly categorized into on-target EGFR-dependent alterations, where tumor cell proliferation remains linked to EGFR signaling, off-target EGFR-independent mechanisms, where other parallel molecular pathways circumvent EGFR signaling, histological transformations and other emerging mechanisms. The differential resistance mechanisms observed when osimertinib is administered in first-line versus second-line settings highlight varying selective pressures and clonal evolution dynamics (Bertoli et al., 2022). This indicates that the initial tumor heterogeneity and subsequent treatment history significantly dictate the emergence of resistance, suggesting that a universal approach to managing resistance is unlikely to be effective. Instead, dynamic molecular profiling and adaptive treatment strategies are essential to address this evolving landscape.

## On-target EGFR-Dependent mechanisms

These mechanisms involve further genetic alterations within the EGFR gene itself, maintaining the tumor’s reliance on EGFR signaling despite TKI therapy.

### EGFR C797S mutation

The C797S mutation is the most common EGFR-dependent resistance mechanism to osimertinib, frequently observed when osimertinib is used either as first-line therapy or as second-line treatment following T790M mutation (Cheng et al., 2025; Zalaquett et al., 2023). This mutation disrupts the covalent bond formation between osimertinib and the Cys797 residue in the ATP-binding site of the EGFR kinase domain, thereby impairing drug binding and leading to reduced sensitivity (Niederst et al., 2015). The prevalence of C797S varies across studies, reported as 6%–26% of cases overall (Gogleva et al., 2022), and 18.1% in one cohort (with 52.2% T790M mutation of the cases retaining) (Cheng et al., 2019). When osimertinib is administered as first-line treatment, C797S is observed in 7%–15% of patients (Leonetti et al., 2019; Chmielecki et al., 2023a; Passaro et al., 2021).

A notable aspect of C797S resistance, particularly when the T790M mutation is lost, is the potential re-emergence of sensitivity to first-generation TKIs (Rangachari et al., 2019). This phenomenon reveals a fascinating evolutionary “loop” in tumor adaptation. Osimertinib effectively targets T790M, but the tumor adapts by acquiring C797S, which then re-sensitizes it to earlier drugs that do not rely on C797 covalent binding. This is a direct consequence of the molecular mechanism: osimertinib’s efficacy hinges on its covalent bond at C797, which is abrogated by the C797S mutation, while first-generation TKIs do not form this covalent bond and are therefore unaffected by C797S alone (Díaz-Serrano et al., 2018). However, this re-sensitization is often transient, as tumors can rapidly re-acquire both T790M and C797S in the *cis* configuration, leading to broad TKI resistance (Rangachari et al., 2019; Wang et al., 2017; Zhou et al., 2019). This highlights the dynamic and complex nature of tumor evolution under selective pressure, where therapeutic interventions drive successive adaptive changes.

### Other novel EGFR mutations

Beyond C797S, other secondary EGFR mutations have been identified that significantly decrease sensitivity to osimertinib. These include mutations such as L792, L718Q, and G796 (Yang et al., 2018; Oxnard et al., 2018; Klempner et al., 2017). These mutations are strategically located in the hinge pocket of the ATP-binding domain, in close proximity to both C797S and T790M, and their presence can directly interrupt osimertinib binding to the receptor (O'Leary et al., 2020).

### EGFR amplification and T790M loss

In patients who received second-line osimertinib for T790M-positive disease, the loss of the T790M mutation is frequently observed at the time of progression, and this loss is associated with earlier resistance and poorer survival (Lee et al., 2021; Vadagam et al., 2025). This often correlates with the development of alternative resistance mechanisms, as the tumor seeks new pathways for survival. Additionally, amplification of the EGFR gene itself can also contribute to acquired resistance to third-generation TKIs (Le et al., 2018; Li et al., 2023a). Studies found EGFR amplification more often occures in acquired resistance cases of osimertinib which was commonly mixed with activating EGFR mutations, so the deep role of EGFR amplication is urgently needed to explore (Knebel et al., 2017; Nukaga et al., 2017; Roper et al., 2020).

## Off-target EGFR-Independent bypass pathway activations

In contrast to on-target mechanisms, off-target resistance involves the activation of alternative signaling pathways that allow tumor proliferation to become independent of EGFR signaling (Crystal et al., 2014; Rotow and Bivona, 2017).

### MET amplification/overexpression

Amplification of MET gene or MET protein overexpression is a prominent off-target resistance mechanism to osimertinib, detected in approximately 25% of osimertinib-resistant NSCLC cases (Yu et al., 2013; Passaro et al., 2021; Angelopoulos et al., 2025). This amplification leads to the activation of downstream signaling pathways, including PI3K/AKT/mTOR and MAPK pathways, effectively bypassing the inhibition of EGFR (Wang et al., 2019; Zhang Y. et al., 2018). Its significance is particularly pronounced in cases where the T790M mutation is lost upon progression (Lee et al., 2021). Besides, increasing studies also found *MET* alterations and acquired *MET* mutations potentially plays vital role in osimertinib resistance (Zhang Y. et al., 2018; McCubrey et al., 2007; Bennett et al., 2016).

### HER2 amplification/mutations and HER3 upregulation

Aberrations in HER2 (ERBB2), including gene amplification, exon 20 insertions, and the D16 splice variant, are important mechanisms of acquired resistance to first-generation TKIs and also emerge following osimertinib treatment. HER2 amplification rates are reported at approximately 5% in second-line and 2% in first-line resistance settings post-osimertinib, often in the absence of T790M mutations (Papadimitrakopoulou et al., 2018; Ramalingam et al., 2018). HER3, a member of the ErbB family that lacks intrinsic kinase activity, plays a pivotal role by dimerizing with other ErbB receptors, particularly HER2, to robustly activate the PI3K/AKT pathway. And upregulation of HER3 is observed in osimertinib-resistant cells, further reinforcing its role as a mediator of resistance (Liao et al., 2025).

### RAS/MAPK pathway alterations

Mutations in key genes within the RAS/MAPK and PI3K/AKT/mTOR signaling pathways, such as KRAS, BRAF, and PIK3CA, can activate downstream signaling cascades independently of EGFR. These alterations contribute significantly to driving resistance to EGFR TKIs (Yang et al., 2018).

### Other receptor tyrosine kinase (RTK) activations

Beyond the commonly recognized bypass pathways, other RTKs, such as FGFR1 and IGF1R, can also become aberrantly activated. Additionally, overexpression of AXL has been observed, providing alternative survival signals that contribute to acquired resistance to third-generation TKIs (Lu et al., 2020; Wang et al., 2020; Taniguchi et al., 2019).

## Histological and phenotypic transformations

Tumor cells can undergo profound changes in their cellular identity, leading to TKI resistance which accounts for 5%–7% and severely relies on repeated biopsy (Lee et al., 2021; Sequist et al., 2011). In patients with EGFR-mutant lung adenocarcinoma, acquired resistance to TKI therapy almost inevitably develops within a timeframe of 18 months. Among the various resistance mechanisms, histologic transformation has been identified as a clinically relevant contributor, documented in up to 14% of cases. hshs enhance the discussion (Banerjee et al., 2025). Histologic transformation in LUAD commonly gives rise to two distinct phenotypes: small cell lung cancer with neuroendocrine features (T-SCLC) and squamous cell carcinoma (T-LUSC) while both histologic type have poorer survival rate compared with LUAD, particularly in advanced stages (Sato et al., 2022). Epithelial-Mesenchymal Transition is another vital mechanism for EGFR-TKI resistance that epithelial tumor cells lose polarity and gain a mesenchymal, apoptosis-resistant phenotype. Furthermore, some studies have also reported rare transformations in which patients with EGFR-mutant LUAD ultimately converted to sarcoma after acquiring EGFR-TKI resistance featured with poor prognosis (Zhu et al., 2024; Hsieh et al., 2019).

### Small cell lung cancer (SCLC) transformation

A critical mechanism of resistance involves the histological transformation of NSCLC adenocarcinoma into small cell lung cancer (SCLC) (Passaro et al., 2021; Yang and Fan, 2024). The persistence of the identical EGFR mutation from LUAD to T-SCLC strongly supports a transdifferentiation origin, as opposed to *de novo* SCLC development, which rarely features such mutations. (Quintanal-Villalonga et al., 2020; Sivakumar et al., 2023). This transformation is often associated with alterations in cell-cycle regulators, including RB1 and TP53 which were most well-studied molecules involved in SCLC, and typically results in a modest and transient response to platinum-etoposide chemotherapy (Yang and Fan, 2024; Wang H. et al., 2025). SCLC transformation is accompanied by widespread molecular and signaling pathway dysregulation. The process is triggered by *RB1* and *TP53* deletion, together with SOX family mutations, PI3K/AKT pathway activation, *MYC/AURKA* amplification and reduced Notch signaling (Yin et al., 2022). A recent study investigated immune landscape within T-SCLC via single cell RNA sequencing and confirm that stem-like cell clusters and interferon-stimulated gene-positive lymphocytes are critically involved in the progression of LUAD transforming into SCLC (Huang et al., 2026). Another study found pathway reprogramming occurs during SCLC transformation, which is driven by tumor cell epigenetic alterations including HDAC10, HDAC1 and DNMT3A, rather than the tumor microenvironment in EGFR-TKI resistance. Transformed SCLC develops immune exhaustion correlated with prior EGFR-TKI treatment duration and they constructed a 4-marker model (BIRC3, COL6A6, CASP12, GATA2) for predicting SCLC transformation, with robust sensitivity and specificity (Li Y. et al., 2024). There is an urgent need to further elucidate the mechanisms underlying EGFR-TKI resistance through SCLC transformation and to identify potential therapeutic targets.

### Squamous cell carcinoma (SCC) transformation

Less frequently, EGFR-positive adenocarcinoma can transform into EGFR-negative squamous cell carcinoma (SCC) following acquired resistance to EGFR TKIs. This poses significant therapeutic challenges due to the altered histological and molecular profile of the tumor (Schoenfeld et al., 2020; Qiu et al., 2025).

### Epithelial-Mesenchymal Transition (EMT)

Epithelial-Mesenchymal Transition (EMT) is a dynamic and reversible biological process where epithelial cancer cells lose their polarity and adhesion, acquiring mesenchymal features that enhance their motility, invasiveness, and significantly contribute to drug resistance (Qin et al., 2021). This transformation is characterized by the downregulation of epithelial markers, such as E-cadherin, and the upregulation of mesenchymal markers, including N-cadherin and vimentin (Cho et al., 2007; De Craene and Berx, 2013; Zhu et al., 2019; Weng et al., 2019).

The identification of specific molecular drivers like PIM1 kinase in EMT-mediated resistance moves beyond a phenotypic description to a mechanistic understanding, providing actionable therapeutic targets. PIM1 kinase has been identified as a promoter of EMT-associated osimertinib resistance. Its upregulation leads to the functional inactivation of glycogen synthase kinase 3β (GSK3β) through phosphorylation. This inactivation, in turn, suppresses the ubiquitin-proteasome degradation of SNAIL and SLUG, which are snail family transcriptional repressors. The resulting stability and accumulation of SNAIL and SLUG proteins facilitate the EMT process and contribute to osimertinib resistance (Zhou et al., 2024). The ability to inhibit PIM1, for example with agents like SGI-1776, can prevent EMT progression and re-sensitize osimertinib-resistant NSCLC cells to osimertinib, both *in vitro* and *in vivo*. This represents a significant advance in overcoming a complex resistance pathway by targeting its underlying molecular machinery.

## Emerging resistance pathways

Beyond the well-established mechanisms, ongoing research continues to uncover novel pathways contributing to TKI resistance.

### Tumor microenvironment (TME) remodeling

The tumor microenvironment (TME), a complex system comprising tumor cells, immune cells, non-immune cells, and non-cellular components, plays a critical role in carcinogenesis and therapeutic response (Lim et al., 2025). Dynamic changes within the TME during TKI treatment are increasingly recognized as being associated with the development of resistance (Ramachandran et al., 2021). This includes significant shifts in the composition and function of immune cells, as well as the involvement of cancer-associated fibroblasts (CAFs) and neutrophils, which can promote tumor EMT, invasion, and therapy evasion (Peng et al., 2025).

Patients with EGFR mutations exhibit immunosuppressive microenvironments. One recent study used digital spatial profiling to investigate NSCLC samples from 25 patients before and after EGFR-TKI treatment. The results showed significantly altered angiogenic pathways and immune cell proportions following osimertinib therapy, characterized by reduced plasma cells and increased macrophage infiltration. The predominant transition was from the interferon-γ-dominant C2 subtype to the lymphocyte-depleted C4 subtype. These findings uncovered the immunomodulatory effects of osimertinib treatment and implied that dual blockade of angiogenesis and PD-(L)1 might be an effective treatment for EGFR-TKI-resistant NSCLC (Kim et al., 2025). Beyond neoplastic cells, the TME is mainly composed of other cells and extracellular elements within the TME which could both play vital roles in EGFR-TKI resistance progress (Zhang et al., 2024). The cellular compartment mainly consists of tumor-associated myeloid cells (TAMCs), tumor-infiltrating lymphocytes (TILs), natural killer (NK) cells, and cancer-associated fibroblasts (CAFs), while the extracellular compartment includes angiogenesis, secreted factors, and the extracellular matrix (ECM). Specifically, EGFR-TKI treatment could inevitably result in drug resistance and relevant TME changes, including immunosuppressive cytokines and exosomes secreted by CAFs and TAMs, as well as the emergence of certain TIL and CAF subpopulations that contribute to EGFR-TKI resistance. Besides, A recent study constructed a comprehensive atlas of the tumor microenvironment (TME) in brain metastatic lesions of lung cancer by integrating single-cell RNA sequencing data, and identified a malignant, therapy-resistant ACTN1^+^ epithelial cell subcluster, suggesting a potential therapeutic target for overcoming intracranial EGFR-TKI resistance (Wu et al., 2026).

### Epigenetic dysregulation

The discovery of epigenetic dysregulation and non-coding RNA (ncRNA) involvement in osimertinib resistance signifies a shift beyond purely genetic mutations, highlighting the plasticity of cancer cells and their ability to adapt through reversible changes in gene expression. Epigenetic changes, particularly histone modifications, contribute to osimertinib resistance (Dawson and Kouzarides, 2012; Goldberg et al., 2007). For instance, upregulation of FGF1, regulated by epigenetic mechanisms, has been observed in osimertinib-resistant cells and clinical samples. Targeting these modifications with bromodomain and extra-terminal domain (BET) inhibitors has shown promise in overcoming resistance (Miyashita et al., 2025). Non-coding RNAs (ncRNAs), including microRNAs (miRNAs), long ncRNAs (lncRNAs), and circular RNAs (circRNAs), also play significant roles in regulating essential signaling pathways and modulating treatment sensitivity, thereby influencing resistance development (Zeng et al., 2024). This opens entirely new therapeutic avenues, such as epigenetic modifiers or ncRNA-based therapies, which could potentially overcome resistance even in the absence of new actionable mutations.

### Metabolic reprogramming

Mutant EGFR could activates the RAS/RAF/ERK and PI3K pathways and promotes metabolic reprogramming, enhancing glucose uptake and lactate production in lung adenocarcinomas (Joshi and Sheikh, 2025). Besides, EGFR mutations in NSCLC were also found to increase glycolysis, a process required for EGFR stabilization (Kim et al., 2018). Osimertinib resistance is associated with complex metabolic reprogramming within cancer cells. This includes enhanced glycolysis, a phenomenon known as the Warburg effect, and altered lipid metabolism (Zhou et al., 2023; Zhang et al., 2025; Cui et al., 2025). A study found that osimertinib-resistant cells survive glucose deprivation by boosting glucose and glutamine uptake and mitochondrial metabolism while Inhibiting these pathways impaired their proliferation and survival. Targeting the metabolic adaptive responses emerges as a promising strategy for resistance (Eltayeb et al., 2024). DPP4 enhances fatty acid uptake via CPT1A and oxidation, and DPP4-MEK-Nrf2 antioxidant signaling preserves mitochondrial function which comprehensively results in osimertinib-resistant state f lung cancer and this provides new strategy against persister cells from metobolic insights (Zhang et al., 2025). Maroni et al. identified newly the pyruvate-acetaldehyde-acetate (PAA) pathway as a key osimertinib resistance driver recently, generating NADPH (essential for reductive biosynthesis) and acetyl-CoA for biosynthesis (supporting lipid biosynthesis). Integrating multi-omics approaches, this study reveals the metabolic network in osimertinib-resistant EGFR-mutant NSCLC, positioning the PAA pathway as a central hub (Maroni et al., 2025a; Maroni et al., 2025b; Xia et al., 2020). This identification of specific metabolic pathways like PAA provides a novel and potentially broad-spectrum vulnerability. Cancer cells’ metabolic flexibility is a known hallmark, but pinpointing specific pathways that become critical for survival under TKI pressure offers new therapeutic targets that could be less prone to rapid resistance compared to direct kinase inhibition. Furthermore, targeted mitochondrial DNA sequencing and whole genome sequencing have revealed coordinated patterns of mutational remodeling in both mitochondrial and nuclear-encoded OXPHOS genes, indicating bioenergetic adaptations that contribute to resistance (Maroni et al., 2024; Chmielecki et al., 2023b),

### Post-translational regulation of EGFR

After translation, EGFR is subject not only to various other post-translational modifications: including S-palmitoylation, S-nitrosylation, and methylation but also to receive different regulation from other molecular which could regulate proliferation, invasion and drug resistance (Seres et al., 2025). FOXO1 posttranslational modifications regulate EGFR-TKI resistance in NSCLC. The HDAC inhibitor depsipeptide increases FOXO1 acetylation, thereby overcoming TKI resistance and effectively inducing apoptosis in resistant NSCLC cells (Xu et al., 2015). Regarding deubiquitylating enzymes, previous studies demonstrated that USP8, USP22, USP13, USP35 and others could promote NSCLC progression via kinds of mechanisms (Qv et al., 2025; Chen et al., 2024; Wu et al., 2019; Wang et al., 2022). In 2013, one study first demonstrated that USP8 inhibition reduces EGFR, ERBB2, ERBB3, and MET levels, blunts RTK signaling, and selectively kills cancer cells without harming normal cells, thereby overcoming gefitinib resistance in NSCLC (Byun et al., 2013). Besides, USP22 deubiquitinates late endosomal EGFR, blocking degradation and enhancing recycling to drive EGFR-TKI resistance in EGFR-mutant lung adenocarcinoma. USP22 silencing reverses resistance *in vitro* and *in vivo*, making USP22 a therapeutic target (Zhang H. et al., 2018). Some other studies also found USP7, USP8, USP32 and USP22 could enhance EGFR signalling and other signalling while further promoting EGFR-TKI resistance (Jiao et al., 2023; Thai et al., 2021; Meng et al., 2024). Post-translational of EGFR, particularly USP mechanisms plays vital role in EGFR-TKI resistance and further research on USP functions may yield clinical breakthroughs.

### Cytoprotective autophagy

Autophagy is an intracellular degradation process and plays a dual role in cancer while the upregulation helps cancer cells avoid apoptosis, promoting progression and drug resistance, notably in acquired EGFR-TKI resistance in NSCLC (Thai et al., 2021; Sharifi et al., 2016; Lyu et al., 2022; Miller and Thorburn, 2021). One study showed that GLMP promotes Osimertinib resistance by regulating RhoA ubiquitination, which activates EMT and late-stage autophagy. Blocking RhoA alone only enhances autophagic initiation, while lysosomal hyperactivity sustains autophagic flux in resistant cells. Therefore, co-inhibiting both RhoA and autophagy effectively overcomes resistance, offering a promising therapeutic strategy (Liang et al., 2025). A recent study demonstrated STC2 enhances autophagic activity and STC2 activates ERK1/2 activation and Beclin 1-related autophagic activity. While inhibiting autophagy reversed the STC2-induced EGFR-TKI resistance *in vitro* and *in vivo*. Thus, STC2 promotes cytoprotective autophagy, and targeting the STC2-ERK/Beclin1 axis may overcome EGFR-TKI resistance (Liu et al., 2026). Another interesting study showed that PD-L1 promotes proliferation and autophagy while suppressing apoptosis via MAPK signaling, which could drive gefitinib resistance. While gefitinib plus pemetrexed synergistically counteracts this in PD-L1-overexpressing cells. Then, they concluded that PD-L1 contributes to primary EGFR-TKI resistance through MAPK-mediated autophagy offering therapeutic targets for LUAD (Li N. et al., 2024). Singh et al. found that 4″-alkyl EGCG derivatives inhibit EGFR (including mutant L858R/T790M) and block AKT/mTOR, but also trigger cytoprotective autophagy in glioblastoma cells. Combining these derivatives with chloroquine to suppress autophagy enhances caspase-3-dependent apoptosis and cytotoxicity which providing a combinatorial strategy to improve EGFR-TKI efficacy in glioblastoma (Singh et al., 2024). Besides, other studies also found that inhibition of autophagy (chloroquine, 3-MA, YC-1) represent a viable therapeutic approach for overcoming both intrinsic and acquired EGFR-TKI resistance in lung adenocarcinoma (Hu et al., 2017; Hu et al., 2021). All the above resistance mechanisms were concluded in Table 2.

**TABLE 2:** Spectrum and frequency of acquired resistance mechanisms to third-generation EGFR TKIs.

Mechanism category	Specific mechanism	Reported prevalence/Frequency	Molecular consequence/Impact	Relevant subtypes/Context
On-Target	EGFR C797S Mutation	6%–26% overall (Gogleva et al., 2022); 7%–15% (1st-line osimertinib) (Leonetti et al., 2019; Chmielecki et al., 2023a; Passaro et al., 2021); 18.1% overall, 52.2% (retained T790M) (Cheng et al., 2019)	Disrupts covalent binding of osimertinib to Cys797; can re-sensitize to 1st-gen TKIs (if T790M lost) (Rangachari et al., 2019)	First-line and second-line osimertinib; often associated with T790M loss (Díaz-Serrano et al., 2018)
​	Other Novel EGFR Mutations (e.g., L792, L718Q, G796)	Approximately one-third of cohort (combined) (Yang et al., 2018)	Decrease sensitivity to osimertinib by interrupting binding in ATP-binding domain (Yang et al., 2018)	Post-osimertinib progression ()
​	T790M Loss	49% (AURA3 trial, 2nd-line osimertinib) (Chmielecki et al., 2023b); 68% (Oxnard et al.) (Oxnard et al., 2018)	Associated with earlier resistance and poorer survival; often linked to alternative mechanisms (Oxnard et al., 2018)	Second-line osimertinib (for T790M-positive disease) (Oxnard et al., 2018)
​	EGFR Amplification	Contributes to resistance (Le et al., 2018; Li et al., 2023a)	Sustains EGFR signaling (Le et al., 2018; Li et al., 2023a)	General resistance mechanism (Le et al., 2018; Li et al., 2023a)
Off-Target	MET Amplification/Overexpression	∼25% (osimertinib-resistant NSCLC) (Yu et al., 2013; Passaro et al., 2021; Angelopoulos et al., 2025)	Activates PI3K/AKT/mTOR and MAPK pathways, bypassing EGFR inhibition (Wang et al., 2019; Zhang H. et al., 2018)	First-line and second-line osimertinib, particularly when T790M is lost (Soria et al., 2018)
​	HER2 Amplification/Mutations	∼5% (2nd-line osimertinib resistance); ∼2% (1st-line osimertinib resistance) (Papadimitrakopoulou et al., 2018; Ramalingam et al., 2018)	HER2 activation, dimerization with HER3, activates PI3K/AKT pathway (Liao et al., 2025)	Post-osimertinib progression (Gomatou et al., 2023)
​	HER3 Upregulation	Levels several times higher in resistant cells (Liao et al., 2025)	Dimerizes with HER2, robustly activates PI3K/AKT pathway (Liao et al., 2025)	Osimertinib-resistant cells (Liao et al., 2025)
​	RAS/MAPK Pathway Alterations (KRAS, BRAF, PIK3CA)	Frequent, specific prevalence varies by study (Yang et al., 2018)	Activate downstream signaling independently of EGFR (Yang et al., 2018)	General resistance mechanism (Yang et al., 2018)
​	Other RTK Activations (FGFR1, IGF1R, AXL)	Contributes to resistance (Lu et al., 2020; Wang et al., 2020; Taniguchi et al., 2019)	Provide alternative survival signals	General resistance mechanism
Transformation	Small Cell Lung Cancer (SCLC) Transformation	Reported (Passaro et al., 2021)	Adenocarcinoma transforms to SCLC; often associated with RB1/TP53 alterations (Yang and Fan, 2024; Wang H. et al., 2025)	Post-osimertinib progression (Yang and Fan, 2024)
​	Squamous Cell Carcinoma (SCC) Transformation	Rare, reported (Schoenfeld et al., 2020; Qiu et al., 2025)	EGFR-positive ADC transforms to EGFR-negative SCC (Qiu et al., 2025)	Post-EGFR-TKI resistance (Schoenfeld et al., 2020)
​	Epithelial-Mesenchymal Transition (EMT)	Significant contributor (Qin et al., 2021)	Epithelial cells acquire mesenchymal features, enhancing motility, invasiveness, and drug resistance (Qin et al., 2021; Zhu et al., 2019; Weng et al., 2019)	General resistance mechanism (Zhu et al., 2019)
Emerging	TME Remodeling	Associated with resistance (Lim et al., 2025)	Shifts in immune cell composition/function; role of CAFs/neutrophils (Peng et al., 2025)	During TKI treatment (Ramachandran et al., 2021)
​	Epigenetic Dysregulation (e.g., FGF1 upregulation)	∼40–50% of mechanisms unknown, epigenetic involvement implicated (Dawson and Kouzarides, 2012; Goldberg et al., 2007)	Reversible changes in gene expression; FGF1 upregulation contributes to resistance (Miyashita et al., 2025)	Osimertinib-resistant cells/clinical samples (Goldberg et al., 2007; Miyashita et al., 2025)
​	Non-coding RNA (ncRNA) Involvement	Significant role (Zeng et al., 2024)	Regulate signaling pathways, modulate treatment sensitivity (Zeng et al., 2024)	Osimertinib resistance (Zeng et al., 2024)
​	Metabolic Reprogramming (e.g., glucose shortage, DDP4, PAA pathway)	Associated with resistance (Zhou et al., 2023; Maroni et al.)	Enhanced glycolysis, altered lipid metabolism, PAA pathway drives NADPH/acetyl-CoA production (Zhang et al., 2025)	Osimertinib-resistant cells (Maroni et al.)
​	Post-translational regulation of EGFR	Promote malignant progression and resistance (Chen et al., 2024; Zhang Y. et al., 2018)	Enhance EGFR signalling, recycling (Zhang Y. et al., 2018; Thai et al., 2021)	Osimertinib-resistant cells (Meng et al., 2024)
​	Cytoprotective Autophagy	Associated with resistance (Liang et al., 2025; Liu et al., 2026; Hu et al., 2017)	Regulate RhoA pathway, activate EMT and autophagy (Liang et al., 2025)	Osimertinib-resistant cells (Liang et al., 2025)

## Diagnostic approaches for resistance mechanisms

**FIGURE 2:**
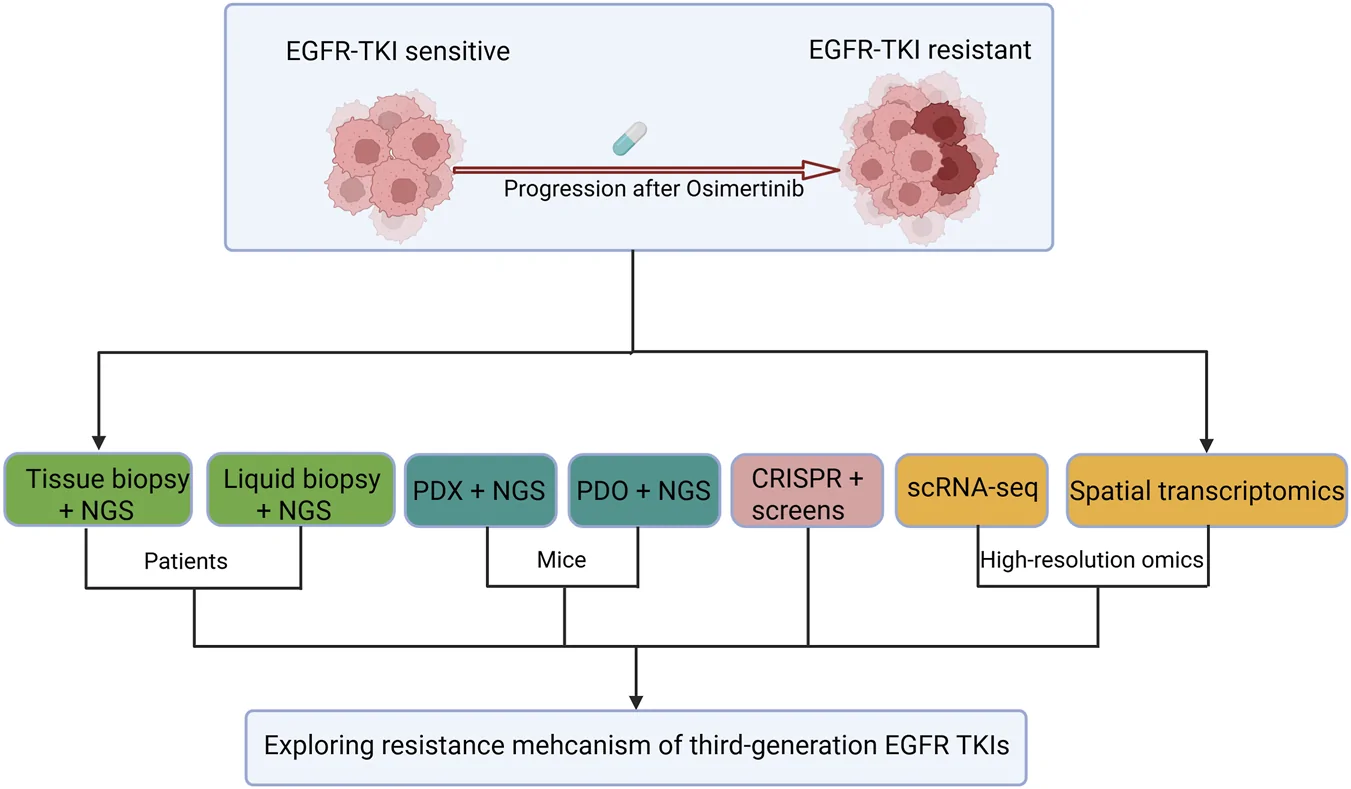
Approaches for investigating resistance mechanisms.

The dynamic nature of tumor evolution necessitates a shift towards comprehensive, dynamic molecular profiling for effective management of acquired resistance. This implies that treatment decisions should ideally be guided by real-time, evolving molecular insights rather than a single baseline biopsy, moving towards a truly adaptive precision oncology paradigm. The current and promising approaches for investigating resistance mechanisms is showed in Figure 2.

### Liquid biopsy (ctDNA) and Next-Generation Sequencing (NGS)

Instead of invasive tissue biopsy, liquid biopsy, primarily through the analysis of circulating tumor DNA (ctDNA), offers a minimally invasive method for real-time monitoring of resistance mechanisms (Zhao et al., 2026). Next-Generation Sequencing (NGS) is the most commonly employed technique for comprehensive genomic profiling of ctDNA, enabling the simultaneous detection of multiple genetic alterations, including tertiary EGFR mutations (e.g., C797S) and the activation of bypass pathways (Ito et al., 2023). Frequent liquid biopsies are valuable for the early detection of disease relapse and for identifying newly emerging driver mutations, providing a dynamic molecular snapshot of the tumor’s evolving landscape.

### Advanced preclinical models

Sophisticated preclinical models are indispensable for dissecting resistance mechanisms and testing novel therapeutic strategies.

### Patient-derived xenografts (PDX)

Patient-derived xenograft (PDX) models are established by transplanting fresh tumor tissues from patients into immunodeficient mice. These models closely mimic the original tumor’s morphology, genetic characteristics, and drug reactivity *in vivo*, making them preferred preclinical tools for evaluating drug sensitivity, exploring mechanisms of resistance (e.g., identifying RAS/MAPK pathway overactivation in osimertinib resistance), and guiding individualized treatment selection (Wang et al., 2024).

### Patient-derived organoids (PDO)

Patient-derived organoids (PDOs) are three-dimensional cell cultures derived from patient tumor tissue that preserve key biological characteristics of the original tumor, including its heterogeneity and drug response (Li et al., 2023b). PDOs offer several advantages over PDX models, including higher culture success rates and significantly reduced time and cost for establishment. These models serve as a promising high-throughput screening platform to identify osimertinib resistance mechanisms and discover novel drug combinations (Naykwadi and Alavala, 2026).

### Functional genomic screens (e.g., CRISPR screens)

CRISPR-Cas9 genome-wide screens, encompassing knockout, interference, and activation modalities, represent high-throughput technologies used to systematically investigate resistance mechanisms (Gogleva et al., 2022). These screens can identify genes that, when altered, either increase cellular sensitivity to a drug (informing potential combination therapies) or drive drug resistance. They are applied in EGFR-mutant NSCLC cell lines treated with TKIs to accurately resemble clinical scenarios of acquired resistance.

### High-resolution omics

Advanced omics technologies provide unprecedented detail in understanding tumor biology.

### Single-cell RNA sequencing (scRNA-seq)

Single-cell RNA sequencing (scRNA-seq) provides single-cell resolution insights into tumor heterogeneity and the dynamic changes within the tumor microenvironment (TME) during the development of EGFR-TKI resistance (Peng et al., 2025). This technology is instrumental in identifying resistance-related cell subpopulations, characterizing their unique molecular features, and discovering novel biomarkers (e.g., SLC40A1-positive macrophages, CD74 upregulation, ASCL1, WNT/β-catenin pathway activation) (Song et al., 2025; Kashima et al., 2021; Hu et al., 2024; Maynard et al., 2020).

### Spatial transcriptomics

Spatial transcriptomics is an innovative approach that unravels the complexities of the TME by capturing mRNA expression data while preserving the spatial relationships between cells in both pre- and post-treatment tumor samples (Spatial Dynamics, 2025). This methodology can reveal the contribution of an inflammatory TME and tumor heterogeneity to the early onset of resistance, allowing for detailed analysis of cell-cell interactions and the spatial distribution of various cell types within the tumor architecture. Table 3 summarized all the advanced methodologies above for resistance discovery and monitoring.

**TABLE 3:** Advanced methodologies for resistance mechanism discovery and monitoring.

Method	Key application in resistance research	Advantages	Limitations/Challenges
Liquid Biopsy (ctDNA/NGS)	Real-time monitoring of resistance mechanisms, early relapse detection, identifying new driver mutations	Minimally invasive, repeatable, provides dynamic molecular snapshots (Zhao et al., 2026)	ctDNA fraction can be low, RNA instability in liquid biopsy, cost (Ito et al., 2023)
Patient-Derived Xenografts (PDX)	Evaluating drug sensitivity, exploring resistance mechanisms, selecting individualized treatments	Preserves tumor morphology, genetic characteristics, and drug reactivity *in vivo* (Wang et al., 2024)	High cost, time-consuming, ethical considerations, requires fresh tumor tissue (Wang et al., 2024)
Patient-Derived Organoids (PDO)	Screening platform for resistance mechanisms, discovering novel drug combinations, drug sensitivity testing	Higher culture success rate, reduced time/cost vs. PDX, preserves tumor heterogeneity (Li et al., 2023b)	Still developing, may not fully replicate *in vivo* TME interactions (Naykwadi and Alavala, 2026)
CRISPR Screens	Identifying genes that drive drug resistance or increase drug sensitivity	High-throughput, systematic investigation of genetic drivers of resistance (Gogleva et al., 2022)^]^	Requires cell line models, results need validation in more complex systems (Gogleva et al., 2022)
Single-Cell RNA Sequencing (scRNA-seq)	Insights into tumor heterogeneity, TME dynamics, identification of resistance-related cell subpopulations and biomarkers	Single-cell resolution, reveals subtle changes in gene expression and cell types (Peng et al., 2025)	High cost, complex data analysis, large data volumes (Peng et al., 2025)
Spatial Transcriptomics	Unraveling TME composition and spatial relationships, understanding inflammatory TME in early resistance	Preserves spatial context of gene expression, comprehensive mRNA capture (Spatial Dynamics, 2025)	Still emerging, complex data analysis, high cost (Spatial Dynamics, 2025)

## Clinical implications and emerging therapeutic strategies

**FIGURE 3:**
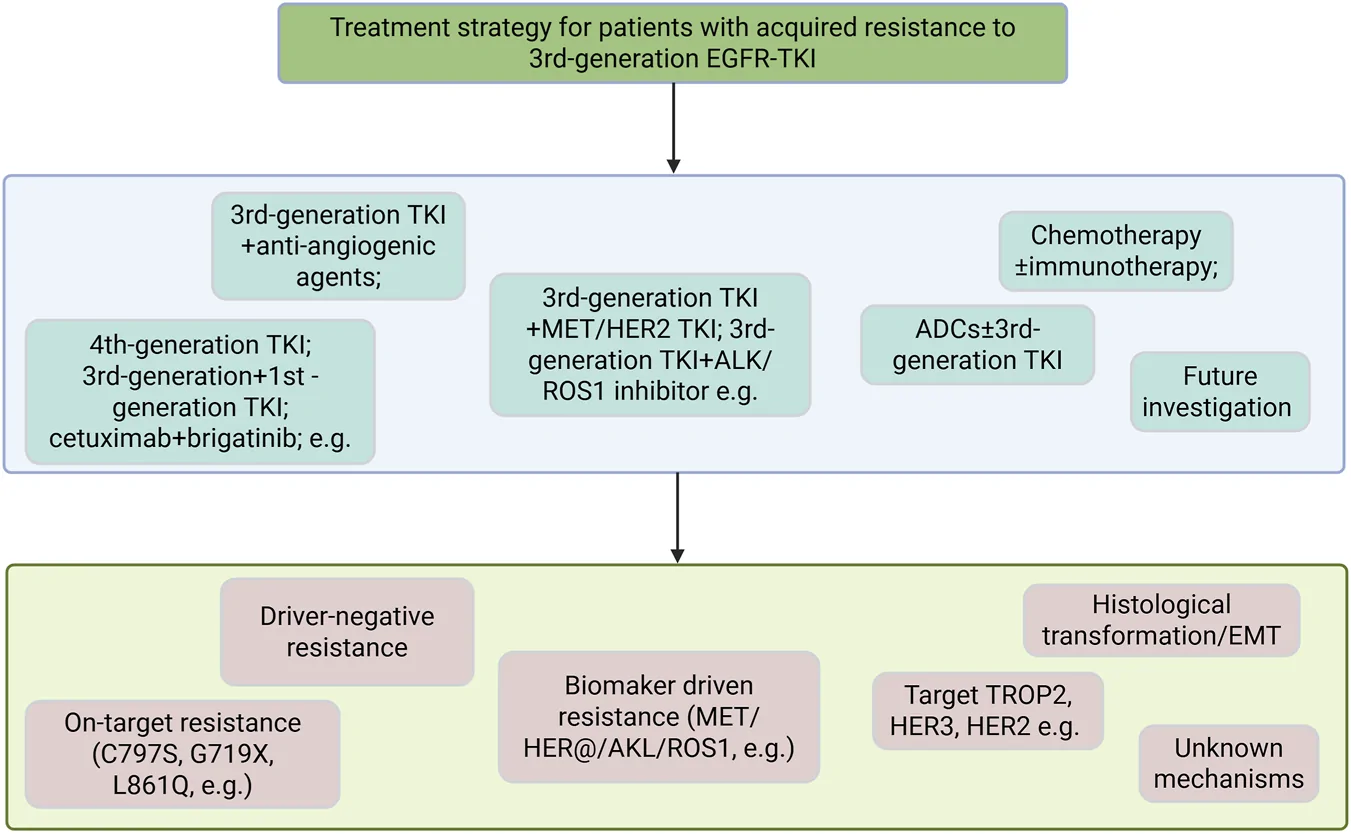
The emerging therapeutic strategies for overcoming resistance.

Despite the remarkable efficacy of osimertinib, disease progression inevitably occurs, and effective treatment options for patients who develop resistance remain a significant unmet need (Gomatou et al., 2023). The consistent exploration of combination therapies across various resistance mechanisms signifies a paradigm shift from sequential monotherapy to multi-targeted or multi-modal approaches. This reflects a growing understanding that tumor heterogeneity and redundant signaling pathways necessitate simultaneous inhibition of multiple vulnerabilities to achieve more durable responses and overcome the inherent adaptability of cancer cells. The emerging strategies aiming overcome resistance is briefly demonstrated in Figure 3.

### Current treatment paradigms for resistant disease

The optimal management strategy after osimertinib progression is an area of active research. Given the complexity and heterogeneity of resistance mechanisms, a personalized approach guided by molecular profiling is increasingly emphasized (Attili et al., 2025).

### Combination therapies

The recognition of diverse resistance mechanisms has spurred the development of various combination strategies.

### Osimertinib with chemotherapy

Combining osimertinib with platinum-pemetrexed chemotherapy has shown promise in delaying the onset of resistance and enhancing control of central nervous system (CNS) metastases. For instance, the FLAURA2 trial demonstrated improved median PFS to 25.5 months with combination therapy compared to 16.7 months with osimertinib monotherapy (Planchard et al., 2023). This strategy aims to combat intratumoral heterogeneity by simultaneously targeting multiple pathways, providing a double therapeutic assault (Schmid et al., 2020).

### Osimertinib with MET/HER2/HER3 inhibitors

Dual EGFR/MET inhibition, such as combining osimertinib with MET inhibitors like savolitinib or the bispecific EGFR/MET antibody amivantamab, has emerged as a promising strategy for MET-driven resistance (Schmid et al., 2020). Clinical trials like TATTON have reported encouraging objective response rates (ORR) and PFS in patients with MET amplification (Hartmaier et al., 2023). Similarly, HER3-targeting strategies, exemplified by the antibody-drug conjugate patritumab deruxtecan (HER3-DXd), show promise in patients who have progressed on EGFR TKIs and platinum-based chemotherapy (Yu et al., 2023a; Yu et al., 2023b).

### Osimertinib with anti-angiogenic agents

Combining osimertinib with inhibitors of the VEGF pathway, such as ramucirumab or bevacizumab, has demonstrated promising extensions in PFS in clinical trials (Yu et al., 2020; Le et al., 2025). This approach targets the tumor’s blood supply, which is critical for growth and metastasis.

### Osimertinib with immunotherapy

Besides the challenge of increased immune-related adverse events, immune-checkpoint inhibitors are generally regarded as having limited efficacy in EGFR-mutant NSCLC patients with acquired resistance to EGFR TKIs (Gara et al., 2020; Hayashi et al., 2022). Research is focused on identifying optimal timing and specific patient populations that might derive benefit from such combinations, particularly given the dynamic changes in the tumor microenvironment induced by TKI treatment (Li et al., 2018; Zhao et al., 2024; Schoenfeld et al., 2019) A recent study found that PD-L1 drive LUAD cell proliferation and autophagy while suppressing apoptosis, promoting tumor progression and gefitinib resistance and PD-L1could be a potential treatment target. Combining gefitinib with pemetrexed synergistically enhanced antitumor efficacy in PD-L1-overexpressing LUAD cells, offering a new approach to overcome EGFR-TKI resistance (Li N. et al., 2024). A clinical trial also showed that, in EGFR-mutant NSCLC following EGFR-TKI therapy, adding atezolizumab to bevacizumab and chemotherapy improved PFS but not OS versus bevacizumab plus chemotherapy (Nogami et al., 2022). The optimal timing and selection of EGFR-mutant patients for immunotherapy combinations warrant further investigation and a wider perspective.

### Antibody-drug conjugates

In HERTHENA-Lung01, HER3-DXd was tested in 225 EGFR-mutant NSCLC patients after TKI/platinum failure (209 post-osimertinib) while osimertinib-resistant patients achieved ORR 29.2%, DCR 72.7%, mPFS 5.5 months, mOS 11.9 months which is a favorable results (Yu et al., 2023b). Randomized phase III HERTHENA-Lung02 compared HER3-DXd vs. pemetrexed-platinum in 586 HER3-unselected EGFR-mutant NSCLC patients post-third-generation EGFR TKI. ORR and PFS significantly favored HER3-DXd, while OS was similar (Mok et al., 2025). In TROPION-Lung01, the first phase III trial of a TROP2 ADC in NSCLC, the TROP2 ADC Dato-DXd yielded a median PFS of 5.7 versus 2.6 months (HR 0.35) with actionable alterations (84 EGFR-mutant) (Ahn et al., 2025a). A pooled analysis of this trial and phase II TROPION-Lung05, involving 117 EGFR-mutant NSCLC patients (96 post-osimertinib), showed a confirmed ORR of 43%, median PFS 5.8 months, and median OS 15.6 months (Ahn et al., 2025b). As for HER2-targeting ADCs, in TRAEMOS trial, T-DM1 plus osimertinib showed limited efficacy in 27 EGFR-mutant NSCLC patients with HER2 overexpression post-osimertinib (ORR 4%, DCR 33%, mPFS 2.8 months) (Jebbink et al., 2023). In another study, HER2 ADC T-DXd achieved 68.4% ORR in 19 HER2-overexpressing EGFR-TKI-pretreated patients (mPFS 8.2 m, mOS 19.6 m) which was favorable (Planchard et al., 2024). SYS6010 is an EGFR-targeted ADC with a TOPO1 inhibitor and showed encouraging efficacy in EGFR-mutant advanced NSCLC in clinical trial (Lu et al., 2025). As EGFR-HER3 bispecific ADCs, BL-B01D1 achieved 67.5% ORR and 87.5% DCR in 41 EGFR-mutant NSCLC after progression from EGFR-TKI(37 treated third-generation TKI), with mPFS 5.6 months indicating promise (Ma et al., 2024). Besides, Peptide-based EGFR inhibitors, PROTAC-based EGFR degraders, Natural-product-derived EGFR inhibitor also have been explored and demonstrated some promising treatment efficacy in EGFR-TKI resistance while needs further investigation and validation (Hallaji et al., 2025; Hou et al., 2026; Zang et al., 2020).

### Novel targeted combinations

Beyond the aforementioned strategies, other emerging approaches include combining osimertinib with PIM1 inhibitors to overcome EMT-associated resistance (Zhou et al., 2024), or with epigenetic modulators like BET inhibitors to target FGF1 upregulation (Miyashita et al., 2025). Aspirin in combination with osimertinib is also being explored for its potential to promote apoptosis and overcome resistance (Han et al., 2020). Patient-derived organoids are proving invaluable in screening for effective drug combinations, leading to the identification of compounds targeting topoisomerases, JAK, PLK1, and PI3K pathways as potential synergistic agents (Yu et al., 2025).

### Patient stratification and personalized medicine

Understanding co-mutational profiles—including alterations in TP53, STK11, KEAP1, PIK3CA, and RB1—is critical for effective patient stratification and tailoring treatment strategies (Attili et al., 2025). These co-mutations significantly influence disease progression, therapeutic resistance, and overall clinical outcomes. Comprehensive genomic profiling through NGS is becoming essential for personalized risk stratification and guiding treatment decisions, allowing for a more precise selection of therapies based on the tumor’s unique molecular landscape.

### Treatment sequencing considerations

Dynamic genetic monitoring at the time of disease progression is crucial for guiding subsequent treatment strategies (Enrico et al., 2022). While certain C797S mutations, particularly those without concurrent T790M, might allow for re-treatment with first-generation TKIs, the rapid re-acquisition of T790M/C797S in *cis* often limits the long-term efficacy of this approach (Rangachari et al., 2019). For histological transformations, such as SCLC or SCC, chemotherapy, sometimes combined with immunotherapy, is considered, though efficacy can be limited, especially for brain metastases (Qiu et al., 2025).

## Challenges and future directions

The persistent challenge of acquired resistance to third-generation EGFR TKIs in NSCLC highlights that current solutions, while improving outcomes, are not definitive. This implies a need for fundamentally new therapeutic paradigms that move beyond incremental improvements in TKI design, potentially focusing on broader cellular vulnerabilities or immune modulation, alongside sophisticated adaptive clinical trial designs.

### Development of next-generation inhibitors and adaptive strategies

The continuous emergence of new resistance mutations (e.g., C797S) and the activation of bypass pathways necessitate the development of next-generation EGFR TKIs and other novel targeted agents that can overcome these new challenges (Wang J. et al., 2025; Du et al., 2021). At present, The EGFR kinase domain contains three binding sites: the ATP site, the allosteric site, and an undruggable inactivation site. Since small molecules cannot target the inactivation site, EGFR TKIs are classified as allosteric, ortho-allosteric and ATP-competitive inhibitors (Ahmad and Patel, 2025). As next-generation inhibitor of EGFR-TKI resistance, Allosteric EGFR inhibitors contains EAI045, JBJ-04–125-02, and EAI045s single-agent activity is limited, likely because EGFR dimerization obstructs its binding; potent cytotoxicity against L858R/T790M/C797S mutant cells only occurs with cetuximab (Jia et al., 2016), while JBJ-04–125-02 acts alone *in vitro* and *in vivo*, with enhanced inhibition by cetuximab or osimertinib (To et al., 2019). Mutant-selective ATP-competitive EGFR inhibitors like BLU-945 (Elamin et al., 2023) and BBT-176 (Lim et al., 2023) have shown insufficient activity, and some other inhibitors are in early clinical trials while further investigation is needed. Further, Li et al. created the first orthoallosteric EGFR inhibitor by merging Vandetanib and EAI045 scaffolds and reached a satisfactory IC50 on EGFR L858R/T790M/C797S (Li et al., 2019). Another study expolred that diethenyl sulfoximine probes modify CDK2 and EGFR and incorporating it into EAI045 produced compound 4, one covalent allosteric EGFR inhibitor overcoming C797S, a lysine-directed strategy (Xu et al., 2025). A recent study identified FL30, a flavone-core inhibitor with nanomolar potency against EGFR L858R/T790M and C797S, and an IC50 matching osimertinib. In EGFR mutant NSCLC models, it selectively suppresses EGFR phosphorylation and cancer growth. Kinetic, modeling, and PIR-SEIRA studies reveal orthosteric binding that induces an inactive-like state. These results highlight FL30’s potential and a strategy combining orthosteric binding with allosteric modulation (Romagnoli et al., 2025). Wittlinger et al. newly investigated ATP-allosteric bivalent inhibitors (AABIs) that selectively inhibit EGFR L858R/T790M and L858R/T790M/C797S mutants by binding to the αC-helix allosteric channel. In contrast, related Type I½ inhibitors prove insufficient against resistant mutants. This selectivity shift defines AABIs as ‘Type V’ bivalent inhibitors (Wittlinger et al., 2024a). Heppner et al. also disclosed bivalent EGFR inhibitors bridging imidazole LN2057 and allosteric DDC4002 via a C-linked amide while compound 6 exhibits IC50 1.8 nM against L858R/T790M/C797S (Wittlinger et al., 2024b). A recent study showed TAS3351, a fourth-generation EGFR-TKI, is a promising candidate for relapsed/refractory NSCLC patients with C797S/T790M mutations and brain metastases (Kasuga et al., 2026). Besides, TAS6417 was found potently inhibits common EGFR mutants (Del19, L858R, T790M) and rarer variants (G719X, L861Q, S768I). It also targets exon 20 insertions while sparing wild-type EGFR, showing superior selectivity and a wider therapeutic window than osimertinib (Remon et al., 2020). As ATP-competitive inhibitors BLU-701 shows strong activity against EGFR-mutant tumors harboring C797S and holds promise for treating or preventing central nervous system metastases (Tavera et al., 2022; Singh et al., 2023), Adaptive treatment paradigms, where therapy is dynamically adjusted based on real-time molecular monitoring, are crucial for tailoring interventions to the evolving tumor landscape. Though numerous challenges remain, next-generation EGFR-TKIs are currently advancing rapidly and their future prospects are highly promising (Cohen et al., 2021).

### Addressing tumor heterogeneity and clonal evolution

Tumor heterogeneity, both between and within tumors, and the dynamic clonal evolution under selective pressure are major drivers of resistance (Laface et al., 2023; Wang Z. et al., 2025). Future therapeutic strategies must explicitly account for this inherent complexity to achieve more durable responses.

### Optimizing combination and sequencing regimens

While combination therapies show considerable promise, optimizing the selection and sequencing of these complex regimens remains a critical challenge. This includes carefully balancing enhanced efficacy with acceptable tolerability and effectively managing the increased risks of toxicity, such as interstitial lung disease associated with osimertinib-immunotherapy combinations (Ahn et al., 2017).

### Improving real-time monitoring and biomarker discovery

Real-time molecular monitoring using liquid biopsy and advanced omics technologies, including single-cell RNA sequencing and spatial transcriptomics, is essential for the early detection of resistant clones, often before clinical progression, and for guiding timely therapeutic adjustments. Further research is needed to identify robust predictive biomarkers that can accurately forecast response to different combination therapies and facilitate precise patient stratification (Peng et al., 2025; Li et al., 2025b).

Furthermore, some valuable trials are currently aiming to address EGFR-TKIs resistance, particularly to third-generation agents. These trials is promising for yielding significantly favorable outcomes for patients, we have added Table 4, which provides a summary of various classes of agents designed to overcome TKI resistance.

**TABLE 4:** Ongoing clinical trial aims to overcome EGFR-TKI resistance in EGFR-mutant NSCLC.

Targets/Drugs	NCT unmber	Targeted enrolment	Treatments	Endpoints
On-target resistance	NCT05920135	24	BBT-207 (fourth generation EGFR TKI)	Phase Ia: RDR; TEAEs Phase Ib: RP2D Phase II: ORR
	NCT05519293	76	H002 (fourth generation EGFR TKI)	Safety, Tolerability, Pharmacokinetic and Preliminary Anti-tumor Activity
	NCT05256290	200	BDTX-1535 (fourth generation EGFR TKI)	DLTs; ORR
Off-target resistance	NCT03944772	247	Third -generation EGFR TKI + MET TKI/first -generation EGFR TKI/anti-EGFR antibody/ALK TKI/RET TKI/chemotherapy/MEK inhibitor/TROP2-targeted ADC	ORR
	NCT06110663	314	Almonertinib + HS-10241	PFS
	NCT04868877	576	EGFR-MET bispecific antibody	MTD; RP2D; ORR
	NCT06452433	91	Third generation EGFR TKI + gumarontinib	ORR
	NCT05498389	115	Osimertinib + EMB-01	MTD, RP2D; AEs; ORR
	NCT04077463	701	Lazertinib; amivantamab; lazertinib + amivantamab; lazertinib + amivantamab + carboplatin–pemetrexed	LTs; AEs; ORR; DOR
	NCT05261399	345	savolitinib in combination with osimertinib	PFS
ADCs	NCT06417814	744	Datopotamab deruxtecan + osimertinib	PFS
	NCT06074588	566	Sacituzumab tirumotecan	PFS; OS
	NCT05338970	586	Patritumab deruxtecan	PFS
	NCT06305754	520	Sacituzumab tirumotecan	PFS; OS
	NCT06465238	30	SHR-A1921 or SHR-A2009	ORR
	NCT05816252	356	sacituzumab tirumotecan + carboplatin	Safety and tolerability; ORR
	NCT04676477	246	Patritumab deruxtecan	DLTs; TEAE; SAE; AESI; ORR
	NCT04042701	115	Trastuzumab deruxtecan + pembrolizumab	DLTs; ORR
	NCT06382116	428	Izalontamab brengitecan	PFS
Combinations with chemotherapy	NCT02098954	40	Erlotinib + gemcitabine–platinum	PFS
Combinations with ICIs	NCT06277674	20	Cadonilimab + anlotinib + pemetrexed	ORR
	NCT04405674	120	Tislelizumab + carboplatin–nab-paclitaxel	PFS
	NCT05132413	561	SHR-1701 + pemetrexed + cisplatin/carboplatin + bevacizumab	PFS; AEs
	NCT06277674	20	Cadonilimab + pemetrexed and anlotinib	ORR
	NCT06334757	46	Serplulimab + bevacizumab + carboplatin– pemetrexed	ORR
Other targets	NCT05601973	60	Amivantamab + lazertinib + bevacizumab	ORR
	NCT05401110	60	Osimertinib + carotuximab	RP2D
	NCT06396065	420	Ivonescimab (SMT112/AK112) Plus Pemetrexed and Carboplatin	PFS, OS
	NCT06363734	32	Osimertinib + dalpiciclib	ORR
	NCT04001777	80	Pelcitoclax (APG-1252) + osimertinib	MTD; RP2D
	NCT06095934	20	Neoantigen peptide vaccine + anti-PD-1 antibody	ORR
	NCT04762199	69	MRX-2843 + osimertinib	MTD; RP2D

SAE, serious adverse events; TEAEs, treatment-emergent adverse events; RP2D, recommended phase II, dose; ORR, objective response rate; DLTs, dose-limiting toxicities; PFS, progression-free survival; MTD, maximum tolerated dose; DOR, duration of response; AE, adverse event; ADC, antibody–drug conjugate; ICIs, immune-checkpoint inhibitors; OS, overall survival; RDR, relative dose reduction.

### Lessons from recent clinical trial failures

Umerous clinical trials, such as SAVANNAH trial (osimertinib + savolitinib), TATTON trial, and CHRYSALIS-2, initially designed to achieve meaningful efficacy have ultimately suffered some issues in patients with disease progression following EGFR-TKI therapy. These setbacks provide important lessons for improving future strategies. The failure of MET-TKI combinations can be attributed to several factors. MET amplification can vary considerably from one tumor region to another and change over time, and without a widely accepted cutoff for MET positivity, it is tough to pick out true responders. Moreover, MET inhibitors are often limited by side effects-peripheral edema being a classic example, so the dosing may not hit the target hard enough. On top of this, MET amplification rarely travels alone; it frequently coexists with other alterations such as KRAS mutations or cell-cycle gene amplifications, and acquired mutations in the MET kinase domain can quickly open escape routes making durable responses elusive.

C797S-targeting drugs face particular challenges. When osimertinib stops working, the C797S mutation almost always appears on the same DNA strand as T790M. This cis configuration twists the ATP-binding pocket in a way that makes it extremely hard to craft a drug that hits both mutants while leaving normal EGFR untouched, so the window between efficacy and toxicity stays narrow. Besides, C797S is seldom the whole story. It typically coexists with MET amplification, KRAS mutations, or even uncharacterized resistance mechanisms, meaning that a kinase inhibitor aimed at a single target is unlikely to rein in the entire tumor. And we should be cautious: liquid biopsies can sometimes overstate the importance of small C797S positive subclones.

With regard to immunotherapy combinations, these lung cancers usually develop in never-smokers, carry a low mutation load, and generate few neoantigens which leaves the immune system largely blind to them. At the same time, oncogenic EGFR signaling builds an immunosuppressive environment rich in regulatory T Cells and myeloid-derived suppressor cells with very few functional CD8^+^T Cells getting in. When PD-L1 does show up, it often signals T-cell exhaustion rather than a real immune attack. Importantly, combining an EGFR TKI with a checkpoint inhibitor brings a disturbingly high risk of pneumonitis, especially with osimertinib. Adding chemotherapy might trigger some immunogenic cell death, but it cannot fundamentally reheat a “cold” tumor, which explains why we have not seen a convincing survival advantage.

### Translational research gaps and unmet needs

Despite significant progress in understanding resistance, a substantial proportion of osimertinib resistance mechanisms (estimated at 40%–50%) remain poorly understood (Leonetti et al., 2019). There is a critical unmet need for effective, well-tolerated treatment options for patients who progress on third-generation EGFR TKIs, particularly for those who have exhausted conventional therapies (Ríos-Hoyo et al., 2022). Future research should focus on precise subtyping of resistance, continuous dynamic monitoring, and the strategic regulation of the immune microenvironment to advance personalized treatment approaches and ultimately improve patient outcomes (Tian et al., 2025). The call for novel, chemotherapy-free combinations underscores that the field is moving towards less toxic, more targeted, and potentially more durable solutions, requiring equally complex and flexible clinical approaches beyond traditional linear trials.
